# Multiple strain infection of *Mycobacterium leprae* in a family having 4 patients: A study employing short tandem repeats

**DOI:** 10.1371/journal.pone.0214051

**Published:** 2019-04-04

**Authors:** Partha Sarathi Mohanty, Avi Kumar Bansal, Farah Naaz, Mamta Arora, Umesh Datta Gupta, Pushpa Gupta, Sandeep Sharma, Haribhan Singh

**Affiliations:** 1 Department of Epidemiology, National JALMA Institute for Leprosy and Other Mycobacterial Diseases, M. Miyazaki Marg, Tajganj, Agra, India; 2 Clinical Division, National JALMA Institute for Leprosy and Other Mycobacterial Diseases, M. Miyazaki Marg, Tajganj, Agra, India; 3 Department of Animal Experimentation, National JALMA Institute for Leprosy and Other Mycobacterial Diseases, M. Miyazaki Marg, Tajganj, Agra, India; St Petersburg Pasteur Institute, RUSSIAN FEDERATION

## Abstract

**Background:**

Leprosy is a slow, chronic disorder caused by *Mycobacterium leprae*. India has achieved elimination of leprosy in December 2005 but new cases are being detected and continue to occur in some endemic pockets. The possible ways of transmission of leprosy is not fully understood and is believed that leprosy is transmitted from person to person in long term contact. Studying the transmission dynamics is further complicated by inability to grow *M*. *leprae* in culture medium and lack of animal models. More than one family members were found to be affected by leprosy in some highly endemic pockets. This study reported the transmission pattern of leprosy in a family having 4 patients.

**Methodology/Principal findings:**

We investigated the transmission of leprosy in a single family having 4 patients using microsatellite typing. DNA was isolated from slit skin smear samples taken from the patients and the isolated DNA were amplified using microsatellite loci TA_11_CA_3_. The amplified products were sequenced using Sanger’s sequencing methods and the copy number variation in the microsatellite loci between strains were elucidated by multiple sequence alignment. The result showed that all the 4 members of the family acquired infection from 3 different strains of *M*. *leprae* from 3 different sources. The elder and middle daughters were infected by same types of strains having the repeat unit TA_13_CA_3_ and could have acquired the infection from social contacts of leprosy cases while the father and younger daughter were infected by strains with the repeat unit TA_12_CA_3_ and TA_11_CA_3_ and could have acquired infection from social contacts_._

**Conclusions/Significance:**

The study suggested that three family members viz, elder daughter, father and younger daughter could be infected by *M*. *leprae* from 3 different sources and the history of the disease and genetic analysis showed that the middle daughter acquired infection from her elder sister in due course of contact. This study implies that the transmission of leprosy not only occurred amongst the house hold members but also has been transmitted from social and neighborhood contacts in long term association with the them.

## Introduction

Leprosy, known as Hansen’s disease, is a chronic infectious disease caused by *Mycobacterium leprae*. In 2008, another species known as *Mycobacterium lepromatosis* was found to cause diffuse lepromatous leprosy (DLL) in human [1–3]. It is highly contagious, but its morbidity is low because a large portion of the population is naturally resistant to this disease. The multiplication rate of *M*. *leprae* is slow, thus increasing the incubation period. Symptoms can take as long as 20 years to appear after infection. Such a long incubation period complicates early diagnosis of leprosy. Untreated Multibacillary (MB) patients shed large number of *M*. *leprae* through upper respiratory tract to the environment and responsible for the continued transmission of leprosy [4].

Different authors have suggested that *M*. *leprae* may be present in the soil, in water, on plants or in various animal species including amoeba, insects, fish, primates and armadillos [4–8]. The role of soil and water in the transmission of leprosy has only been speculated upon; it is yet to be recognized and supported by experimental proof. The exact mode of transmission of leprosy is ambiguous. It was thought that leprosy is transmitted through aerosol route of nasal secretions in long term close contact [9]. Furthermore the inability to grow the *M*. *leprae* in the synthetic culture media complicates the detection of leprosy. Molecular detection of leprosy through RLEP-PCR is a useful tool in case detection of leprosy both in PB (Paucibacillary) and MB cases [10]. Like other tools RLEP-PCR is able to detect leprosy bacilli but unable to correlate findings in epidemiological front. Strain typing is a useful tool for tracing the transmission dynamics of bacterial pathogens for which tools like serotyping, phage typing, antibiogram and MLST (Multi locus sequence technology) are being used [11–14]. In case of *M*. *leprae* these tools are ineffective as the bacterium is unculturable. Elucidation of strain differentiation is limited due to availability of small quantity of DNA and conserved nature of the *M*. *leprae* genome [15]. To trace out the transmission dynamics of *M*. *leprae* and to correlate its epidemiological aspects, the analysis of short tandem repeats (STRs) are constructive tools as different types of strains have variable number of repeating units in the tandem repeat region. In the present study we report the occurrence of leprosy in a family by multiple strains of *M*. *leprae* using a novel short tandem repeat sequence analysis.

## Methods

### Ethical statement

The study has been approved by Institutional Ethical Committee (IEC), National JALMA Institute for Leprosy and Other Mycobacterial Diseases, M. Miyazaki Marg, Tajganj, Agra, India, before initiation of the study. Inform consent was taken from each of the patients at the time of sample collection. Participants who were children, informed consent was obtained from a parent/guardian. For this study written informed consent were taken from patients and parent.

### Patient recruitment

The recruitment of patients was done over a period from November 2013 to March 2015. The four patients within the family were diagnosed at different points of time. This was the only family which was identified to be having multiple patients in that village. To elucidate the transmission pattern of leprosy 5 social contacts having leprosy were identified and were included in the study. It was considered that STR is a useful tool to understand the strain variation among the family members, their neighbours and social contact. Detailed clinico-epidemiological information was obtained from each of the patients including clinical presentation of the present event; past history of the disease; history of contact with leprosy patients; demographic information; time of diagnosis; probable date of onset of disease and any history of previous treatment.

### Sample collection

Slit skin smears (SSS) samples of the patients were taken for AFB as per the standard method, and punch biopsies were taken and put in labelled screw capped 2 ml centrifuged tube containing TE buffer. Tubes were labelled for patient ID and transported to the laboratory and stored at -20°C for further processing/experimentation. All the samples were collected in different time period as they were diagnosed as leprosy in different time period.

### Extraction of *M*. *leprae* genomic DNA

DNA was isolated from the tissue samples of the patients following a procedure as adapted by [16]. Briefly bacilli were disrupted by freezing and thawing, followed by enzymatic disruption by lysozyme and proteinase K. Deproteinization was done with chloroform-isoamyl alcohol (24:1 v/v). After a brief centrifugation at 8000 *g* for 5 minutes, the upper phase was collected. DNA was then precipitated with 0.6 volume of isopropanol, washed with chilled ethanol, dried and re-suspended in 20 μl of (TE) Tris-EDTA buffer before being used for PCR amplification.

### Pathogen confirmation by RLEP-PCR

To rule out the occurrence of leprosy by *Mycobacterium lepromatosis* RLEP-PCR was conducted for all the 9 patients. RLEP is the repetitive sequence of *M*. *leprae* genome and is present in 36 copies. RLEP is highly sensitive and specific assay for detection of *M*. *leprae* and represents a sensitivity of 100% for multibacillary patients and 84.6% sensitivity for paucibacillary [17]. RLEP-PCR reactions were performed in 25 μl of reaction mixture consisting of 5 μl of DNA template, 0.2 m mol dNTPs, 0.5 mol primers and 1U taq DNA polymerase. The 129 base pair fragment of RLEP sequence was amplified by using the primers 5’TGCATGTCATGGCCTTGAGG3’ (forward primer) and 5’CACCGATACCAGCGGCAGAA3’ (reverse primer) [18]. PCR was performed using temperature cycles as 95°C for 2 minutes (initial denaturation) followed by 94°C for 30 seconds (denaturation), 58°C for 2 minutes (primer annealing), 72°C for 2 minutes (extension), final extension of 72°C for 8 minutes for 45 cycles and finally kept at 4°C after completion of 45 cycles. Amplification products were resolved in 2% agrose and viewed under gel documentation system.

### Amplification and sequencing of short tandem repeats

To depict the variability among strains 2 STRs viz., GT_6_ and TA_11_CA_3_ region were amplified. All PCR reactions were carried out in 50 μl total volume with 100 pmol of each primer and approximately 20 ng of template DNA. PCR reactions were performed on a Master cycler gradient using the primers 5’CCTATCGATCTATGGCTTCC3’ (forward) and 5’CCCGTACTTTATCGGCTCTA3’ (reverse) for the STR region TA_11_CA_3_ and 5’ CTGATCATAGCCACCAGTGT3’ (forward) 5’GTTAGGTCGAGACCACACAA3’ for GT_6_ region [19]. Reaction conditions were as follows: initial denaturation for 2 min at 96°C, followed by 45 cycles of 1 min at 96°C, 1 min at 58°C for TA_11_CA_3_ and 55°C for GT_6_ and 2 min at 72°C. A final elongation step of 10 min at 72°C was included. All PCR products were sequenced directly using the forward primer in ABI-3130 XL Genetic analyzer. All the PCR experiments were done on stored DNA samples.

### BLAST search, sequence alignment and data analysis

Sequences were BLAST searched for identification of organism and to elucidate the gene of occurrence of the STR. Multiple sequence alignment was carried out using MEGA 4.0 software to assess the variability in the copy number of the STRs among the strains of *M*. *leprae* along with the reference strain of *M*. *leprae* TN strain (AL450380).

## Results

### Case detection

The family comprised off father, mother one son and three daughters. Of the 4 cases father and one middle daughter (D2) were concurrently diagnosed in November 2013 during a house to house survey and the other 2 daughters (D1 and D3) were diagnosed concurrently at the OPD of Model Rural Health Research Unit (MRHRU), Ghatampur, Kanpur in March 2014. The father was MB case while D2 was a PB case. D1 and D3 were both MB cases. On the Ridley-Jopling classification the index was borderline lepromatous leprosy, the father, D1 and D3 were BL while D2 was BT leprosy cases. Out of 5 social contacts 4 were MB and 1 was PB leprosy patients. We designate the social contacts as SC1, SC2, SC3, SC4 and SC5.

#### Case 1

Case 1 is the head of the family and father of all rest three cases. Patient was a MB case and fell in the borderline lepromatous (BL) leprosy. Diffused infiltration was found all over the body having rough surface, ill-defined margin and no oedema. Both ulnar, lateral popliteal and posterior tibial nerves were thickened. No abscess and disabilities were reported from any part of the body. No significant findings were reported in general and systemic physical including eyes, mouth, larynx. Bacteriological index from slit skin smear sample (BI) was found to be 5+.

#### Case 2

Case 2 is the middle daughter (D2) of the first case. Patient was a PB case of leprosy and fell in borderline tuberculoid (BT) leprosy. One hypo pigmented patch was found in the left hand. Patch was well defined, smooth and having flat lesions. No oedema was found. Both the ulnar nerves were thickened and no abscess and disabilities were reported from any part of the body. No significant findings were reported in general and systemic physical including eyes, mouth, larynx. Bacteriological index from slit skin smear sample (BI) was found to be negative.

#### Case 3

Case 3 is the younger daughter (D1) of first case. Patient was a MB leprosy case and fell in borderline leprosy (BL). More than 20 hypo pigmented active patches were found on all over the body including face. Some of the patches were raised and well defined while some were ill-defined. No oedema was found. Both ulnar, lateral popliteal and posterior tibial nerves were thickened. No significant findings were reported in general and systemic physical including eyes, mouth, larynx. Bacteriological index from slit skin smear sample (BI) was found to be 3+.

#### Case 4

Case is the elder daughter (D3) of first case. Patient was a MB leprosy case and fell in borderline leprosy (BL). Eleven hypo pigmented active patches were found on all over the body including face. All the patches were smooth and well defined. Both ulnar, lateral popliteal and posterior tibial nerves were thickened. No significant findings were reported in general and systemic physical including eyes, mouth, larynx. Bacteriological index (BI) from slit skin smear sample was found to be 3+.

The 5 social contacts were categorized as 4 MB and 1 PB leprosy patients. BI of SC1 was 2+ while BI of SC2, SC4 and SC5 were 3+ and SC3 was found to be negative for AFB.

### Pathogen conformation by RLEP-PCR

All the 9 strains of *M*. *leprae* including 4 family members and 5 social contacts were amplified by RLEP-PCR and confirmed by the presence of 129 base pair bands on 2% agarose gel.

### Sequencing, BLAST search and sequence analysis

**Fig 1:**
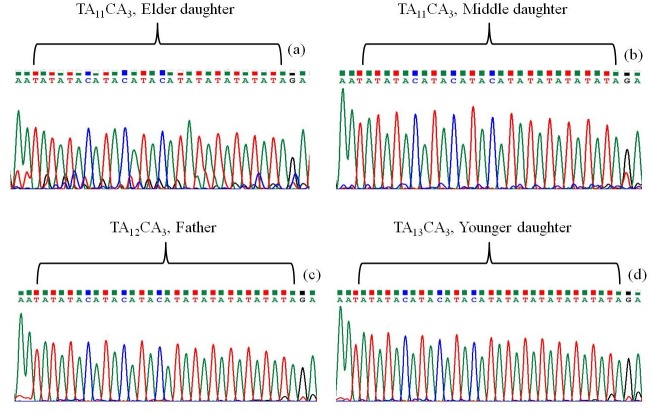
Chromatograph of microsatellite sequence showing copy number variation in different strains of *M*. *leprae*. Microsatellite sequences of *M*. *leprae* found in (a) Elder daughter (TA_11_CA_3_), (b) Middle daughter (TA_11_CA_3_), (c) Father (TA_12_CA_3_), (d) Younger daughter (TA_13_CA_3_).

The STR locus TA_11_CA_3_ was amplified and sequenced for 9 strains (including 5 social contacts) of *M*. *leprae*. The STR locus is present at the flanking region of hypothetical protein (ML0009) and hypothetical protein pseudogene (ML0010). The unambiguous length of the amplified STR region varied from 328 to 332 base pairs. The sequences were submitted to GenBank under the accession numbers KT001966 to KT001969 (family affected with leprosy) and MG762752 to MG762756 (social contacts affected with leprosy). BLAST search clearly differentiated 9 strains into *M*. *leprae*. From the alignment of 4 sequences of family members we found that the copy number of dinucleotide repeat TA varied from 11 to 13 (Fig 1A–1D). The uniqueness of the microsatellite loci was elucidated from the BLAST search and it was found that the satellite region is found only in *M*. *leprae* and the locus was not present in any other group of bacteria whose genes were available in the public data bases like NCBI, EMBL, DDBJ etc except for *Ureaplasma parvum* serovar 3 and *Cyanomargarita calcarea*. On the basis of STR sequence analysis it was found that 3 different types of strains were cause for the leprosy in that particular family. STR repeating unit of TA_11_CA_3_ was found in the strains collected from elder and middle daughter designated as (D3) and (D2) while TA_12_CA_3_ found in the father (F) and TA_13_CA_3_ in the younger daughter (D1) (Fig 1A–1D).

**Fig 2:**
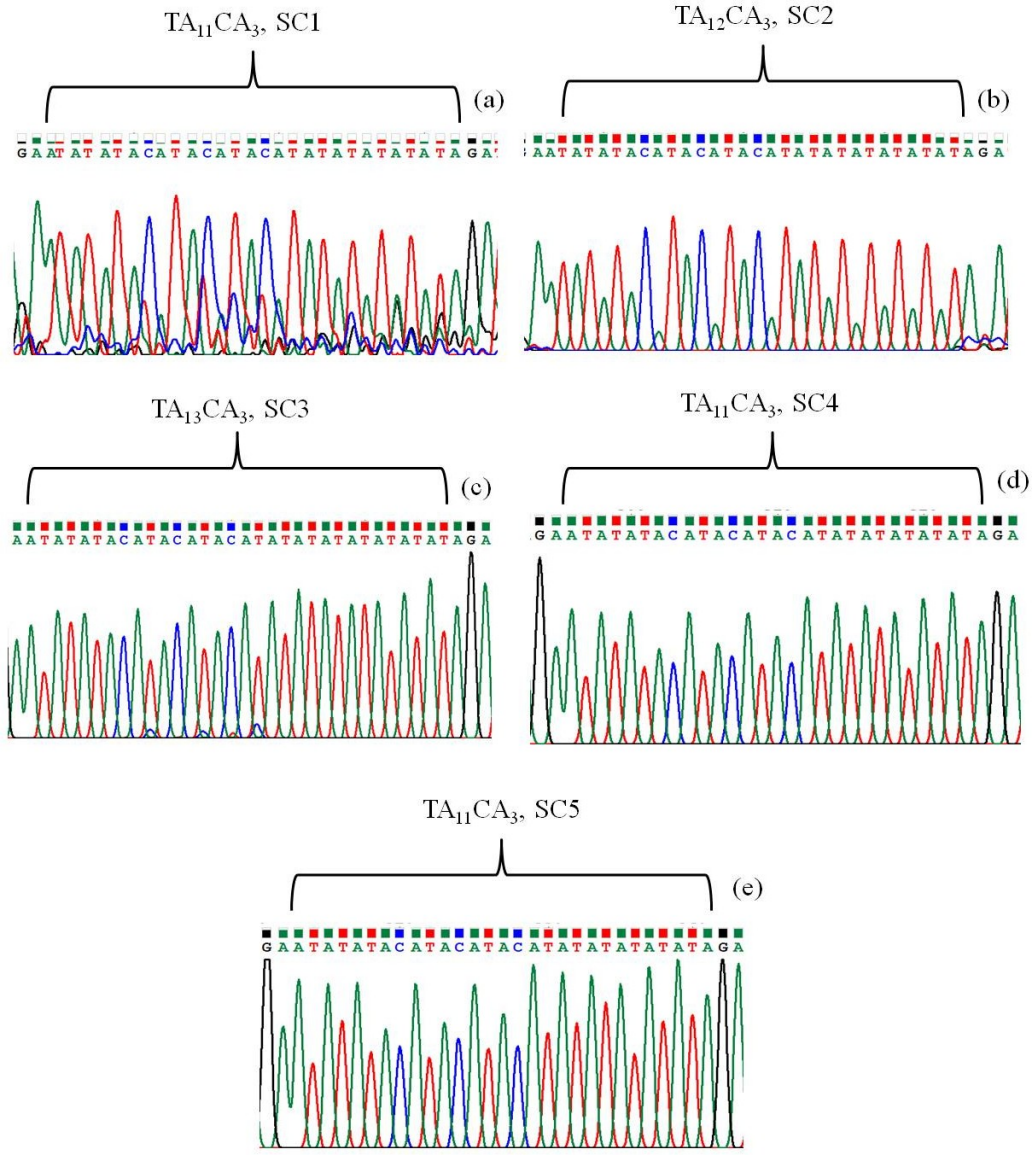
Chromatograph of microsatellite sequence showing copy number variation in different strains of *M*. *leprae*. Microsatellite sequences of *M*. *leprae* found in (a) SC1 (TA_11_CA_3_), (b) SC2 (TA_12_CA_3_), (c) SC3 (TA_13_CA_3_), (d) SC4 (TA_11_CA_3_), (e) SC5 (TA_11_CA_3_).

In case of social contacts the copy number variation observed from 11 to 13. Copy number of dianucleotide TA found to be 11 (TA_11_CA_3_) in SC1, SC4 and SC5, in SC2 it was 12 (TA_12_CA_3_) and in SC3 it was 13 (TA_13_CA_3_) (Fig 2).

**Fig 3:**
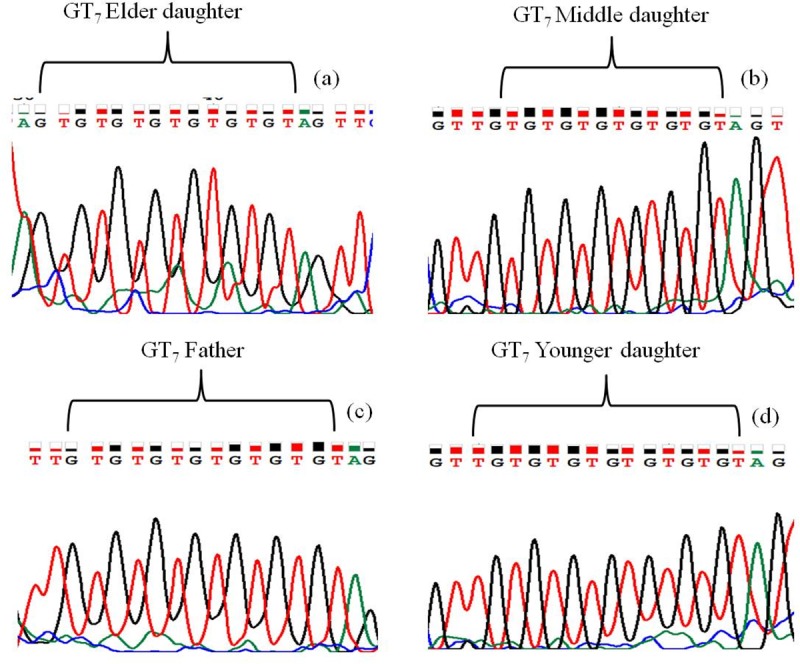
Chromatograph of microsatellite sequence showing conservation of copy number in 4 strains of *M*. *leprae*. Microsatellite sequences of *M*. *leprae* found in (a) Elder daughter GT_7_, (b) Middle daughter GT_7_, (c) Father GT_7_, (d) Younger daughter GT_7_.

We also amplified and sequenced GT_6_ STR region to trace out molecular variability between the 4 strains of *M*. *leprae* found to infect the family members of the particular family. STR region GT_6_ is located in the flanking sequence of conserved protein (ML1825) and cobinamide kinase pseudogene (ML1826). We were unable to observe any copy number variation between the 4 *M*. *leprae* strains of the same family. The observed copy number in the STR region was found to be 7 (Fig 3).

## Discussion

Leprosy is still a public health problem in several countries including India. India achieved elimination target at National level with a prevalence of 0.83/10,000 population in 2005. However, leprosy is still endemic in some pockets of India and new cases are being detected continuously signifying the active transmission of the disease. The disease is said to be transmitted from person to person by respiratory secretions from infected individuals and this theory has not been established so far. Therefore, understanding the transmission dynamics of leprosy is an indispensable element for appropriate intervention strategies for eradication of leprosy.

In last decades several molecular techniques have been used to elucidate the variation among *M*. *leprae* strains either collected from same region or from different region. These molecular techniques mainly based on amplification and sequence analysis of different genes like TTC repeats, rpoT gene, VNTRs and STRs [20–25]. Strain typing using short tandem repeats was widely used for pathogenic bacteria [26–30] and the genome of *M*. *leprae* contains more than 200 tandem repeats including minisatellites and microsatellites. In the present study we used 2 STR loci TA_11_CA_3_ and GT_6._ We found that STR locus TA_11_CA_3_ is unique to *M*. *leprae* to elucidate the transmission dynamics of *M*. *leprae* in a high endemic population. The copy number of GT_6_ STR locus is conserved in all the 4 strains of *M*. *leprae*. In this study 4 members of a family found to be infected with 3 types of *M*. *leprae* strains. Earlier studies illustrated the existence of same type of strain in a family having multiple leprosy cases [31, 32]. In this study the result was relatively unexpected as the multiple cases of the same family acquired infection from multiple strains of *M*. *leprae*. The results were opposite to the earlier research and belief. Such type of situation of leprosy could be due to high endemicity of leprosy where every family had a leprosy patient in last 15 years. It could be due to the short range transmission of the disease from neighbours or from social contacts. Another explanation for the multiple strain infection in a particular family could be due to the evolution of distinct strains of *M*. *leprae* within the index case as a subpopulation expansion [33]. Our study supports the explanation as all the social contacts studied had copy number variation between 11 to 13. It could be possible that elder and middle daughter might be infected from the social contact SC1, SC4 and SC5 as they had the same repeating unit of copy number. Younger daughter might acquire the infection from SC3 because of the same copy number of repeating unit was observed in D1 and SC3. Although the repeating unit of copy number of father and SC2 is same but it still not clear that from where father acquired the infection. At the time of investigation we found that father had no social interaction with SC2. Father might acquire the infection from the social contact of another village or work place. We conclude that the 3 members of the family acquired *M*. *leprae* infection from 3 different sources and the middle daughter acquired infection from her elder sister in due course of contact. The study also reflects the changing pattern of epidemiology of leprosy in an endemic setting in respect to where, whom and how leprosy is transmitted in an endemic area. Finally the study indicated that the leprosy can be acquired from an infected person to a healthy person in long term contact and the transmission can spread via household contacts, neighbourhood contacts as well as other social contacts. Thus all type of contacts are equally responsible for transmission of the disease in a general population where the leprosy is endemic.
